# Accuracy and Effectiveness of Point-of-Care Ultrasound for Peripheral Intravenous Access in Emergency and Trauma Patients: A Systematic Review

**DOI:** 10.7759/cureus.106011

**Published:** 2026-03-27

**Authors:** Latifa Mohammed Jamal Eltyb, Israa Mohamed Ahmed Dafaalla Abbas, Elsheimaa Mohamed Salih, Rim Mohammed Jemli, Sami M. A. Mustafa, Duaa Elshafei Elbadawi Yunis, Nadir Fadol

**Affiliations:** 1 Emergency Department, Wexham Park Hospital, Slough, GBR; 2 Emergency Department, Kosti Teaching Hospital, Kosti, SDN; 3 Emergency Medicine Department, University Hospital Limerick, Limerick, IRL; 4 Emergency Department, King Faisal Medical Complex, Taif, SAU; 5 Accident and Emergency Department, Colchester General Hospital, Colchester, GBR; 6 Emergency Department, Fairfield General Hospital, Northern Care Alliance NHS Foundation Trust, Manchester, GBR; 7 Emergency Department, Croydon University Hospital, London, GBR

**Keywords:** difficult intravenous access, emergency department, peripheral intravenous access, pocus, point-of-care ultrasound, systematic review, trauma

## Abstract

Peripheral intravenous (PIV) access is essential in emergency and trauma care, but challenging in patients with difficult intravenous access (DIVA). This systematic review evaluates the accuracy and effectiveness of point-of-care ultrasound (POCUS)-guided PIV access compared to conventional landmark techniques in emergency and trauma patients. A Preferred Reporting Items for Systematic Reviews and Meta-Analyses (PRISMA)-guided systematic search of PubMed/MEDLINE, Scopus, Web of Science, and Embase was conducted for studies published between January 2021 and December 2025 to focus on contemporary operator training and device improvements, while acknowledging potential time-lag bias. Randomized controlled trials (RCTs) and comparative observational studies reporting first-attempt success, overall success, time to cannulation, attempts, or complications were included. Two reviewers independently screened studies and extracted data, with conflicts resolved by consensus; inter-rater agreement was calculated where possible. Risk of bias was assessed using the Cochrane Risk of Bias 2 (RoB 2) tool and the Risk Of Bias In Non-randomized Studies - of Interventions (ROBINS-I) tool. Nine studies (1,576 participants) met the inclusion criteria: four RCTs, one quasi-experimental, two retrospective cohorts, one prospective observational, and one pilot study. POCUS-guided PIV access significantly improved first-attempt success and overall success (82.4-100% vs. 13.89%, noting the comparator arm may reflect highly selected DIVA patients or less experienced operators) compared to conventional techniques. Fewer attempts were required with POCUS. Time to cannulation was measured from skin contact to successful insertion, including multiple attempts, and was operator- and setup-dependent. Complication rates were comparable or lower with POCUS (definitions included infiltration, extravasation, arterial puncture, and infection; follow-up duration was specified in each study). POCUS approach considered standardized measurement (short-axis vs. long-axis) and vein depth; vessels ≥3.5 mm predicted higher success (OR 2.88, 95% CI 1.39-5.96). Training programmes involved nurses, residents, and attendings; competency was objectively assessed, with some studies using cumulative sum (CUSUM) analysis, and proficiency was achieved after a median of 11 insertions, with post-credentialing success >90% and reduced midline catheter utilization. Outcomes were prospectively measured, minimizing institutional practice drift. Risk of bias was low in five studies, moderate in two, and low/some concerns in two RCTs. POCUS-guided PIV access significantly improves success rates and reduces attempts in emergency and trauma patients with difficult access, with a favourable safety profile. Training diverse operators achieves high proficiency and reduces invasive device use. Findings support routine POCUS use for DIVA patients in acute care settings.

## Introduction and background

Peripheral intravenous (PIV) access is one of the most commonly performed procedures in emergency and trauma care, serving as a critical gateway for fluid resuscitation, medication administration, blood transfusion, and diagnostic testing [1]. In high-acuity environments such as the emergency department and trauma bays, rapid and reliable vascular access is essential to prevent delays in life-saving interventions. However, achieving successful PIV access can be challenging, particularly in patients with difficult intravenous access (DIVA), defined variably in the literature as ≥2 failed attempts, elevated adult difficult intravenous access (A-DIVA) scores, history-based criteria, BMI thresholds, or other institutional definitions, including those with obesity, chronic illness, edema, hypovolemia, intravenous drug use history, or repeated prior cannulations [2]. Multiple failed attempts not only delay treatment but are also associated with increased patient discomfort, procedural complications, higher resource utilization, and the potential need for more invasive alternatives such as central venous catheterization, rescue central access, or midline catheter conversion [3].

Traditionally, PIV cannulation has been performed using landmark and palpation-based techniques. While effective in many patients, the conventional approach relies heavily on visible or palpable veins and operator experience, which may be limited in emergency and trauma populations [4]. In recent years, point-of-care ultrasound (POCUS) has emerged as a valuable bedside tool across various domains of acute care, including trauma assessment, cardiac evaluation, and procedural guidance [5]. Its application in vascular access has gained considerable attention, particularly following the widespread integration of POCUS into emergency medicine practice guidelines and training curricula promoted by organizations such as the American College of Emergency Physicians (ACEP) 2023 ultrasound policy statement.

Ultrasound-guided PIV access enables real-time visualization of veins, surrounding structures, and needle advancement, potentially increasing first-attempt success rates and overall cannulation success while reducing complications such as arterial puncture and infiltration [6]. Several individual studies have compared POCUS-guided PIV insertion with conventional landmark techniques in emergency and trauma settings, reporting varying results in terms of first-attempt success, time to successful cannulation, number of attempts, need for rescue central venous access or midline conversion, catheter dwell time, and patient satisfaction [7]. Some investigations suggest significant benefits in patients with DIVA, whereas others report modest or context-dependent improvements, highlighting ongoing uncertainty regarding its overall effectiveness as a procedural intervention rather than a diagnostic test, across different populations, operator experience levels, and resource settings [8].

Despite the growing body of primary research, findings remain heterogeneous due to differences in study design, patient populations (including trauma versus non-trauma emergency patients), operator training, ultrasound techniques (static versus dynamic guidance), outcome definitions, and implementation feasibility in high-resource versus low-resource settings, including cost-effectiveness considerations. Furthermore, as ultrasound devices have become more portable and affordable, their integration into routine emergency care has accelerated, underscoring the need for a comprehensive synthesis of contemporary evidence [9].

Given the clinical importance of timely vascular access in emergency and trauma patients and the increasing adoption of POCUS-guided techniques, a systematic evaluation of its procedural effectiveness and insertion success, rather than “diagnostic accuracy,” compared with conventional methods, is warranted. Therefore, this systematic review aims to critically appraise and synthesize the available evidence on the effectiveness of POCUS-guided PIV access in emergency and trauma patients, with particular emphasis on first-attempt success rates, overall cannulation success, time to access, complication rates, catheter dwell time, need for midline conversion, and patient-centered outcomes. By consolidating current evidence, this review seeks to inform clinical practice, guide training strategies, and support evidence-based decision-making in acute care settings.

## Review

Methodology

Study Design and Protocol

This systematic review was conducted in accordance with the Preferred Reporting Items for Systematic Reviews and Meta-Analyses (PRISMA) 2020 statement [10]. The methodology was predefined prior to study selection to ensure transparency, reproducibility, and methodological rigor. The review process, including literature search, screening, eligibility assessment, data extraction, and risk of bias evaluation, followed PRISMA recommendations to minimize selection bias and enhance the reliability of findings. This review was not registered in PROSPERO due to the time-sensitive nature of the topic and the rapid evidence synthesis needs.

Eligibility Criteria (PICOS Framework)

Eligibility criteria were defined using the PICOS framework, covering population, intervention, comparator, outcomes, study design, and publication characteristics. The detailed inclusion and exclusion criteria used for study selection are presented in Table 1.

**Table 1:** Eligibility Criteria Based on the PICOS Framework

PICOS Element	Inclusion Criteria	Exclusion Criteria
Population	Adolescent (≥12 years) or adult patients (≥18 years) presenting to emergency departments or trauma settings requiring peripheral intravenous (PIV) access, including patients with difficult intravenous access (DIVA). Operator types included nurses, residents, and attending physicians.	Pediatric-only populations (<12 years) or studies conducted exclusively in non-acute care settings without emergency or trauma populations.
Intervention	Point-of-care ultrasound-guided peripheral intravenous cannulation (static or dynamic ultrasound guidance). Multiple failed landmark attempts prior to crossover were allowed if reported; maximum attempt number noted where standardized.	Studies not involving ultrasound guidance or evaluating other vascular access techniques only.
Comparator	Conventional landmark-guided peripheral intravenous cannulation. Comparator operator experience equivalent to intervention arm where reported.	Studies without a comparator group.
Outcomes	First-attempt success rate (defined as successful cannulation with a single skin puncture without needle redirection), overall success rate, time to cannulation (measured from skin contact or decision-to-access as reported), number of attempts, complications, need for alternative vascular access, patient satisfaction, pain scores, catheter dwell time.	Studies not reporting procedural success or clinically relevant outcomes.
Study Design	Randomized controlled trials and comparative observational studies. Trauma-only studies analyzed separately where possible to reduce clinical heterogeneity.	Case reports, case series, narrative reviews, systematic reviews, editorials, letters, grey literature and conference abstracts. Excluding abstracts may increase publication bias.
Publication Period	Studies published between January 2021 and December 2025.	Studies published before 2021.
Language	Articles published in English.	Non-English publications without accessible translations.

Information Sources

A comprehensive literature search was conducted across the following electronic databases: PubMed/MEDLINE, Scopus, Web of Science, and Embase (Elsevier). These databases were selected to ensure broad coverage of biomedical, clinical, and interdisciplinary research relevant to emergency medicine and ultrasound-guided procedures. The Cochrane Central Register of Controlled Trials (CENTRAL) was not searched because the included studies were primarily procedural and observational in nature, and CENTRAL focuses mainly on randomized controlled trials (RCTs) in general clinical interventions. After the initial screening, an updated final search was conducted on February 22, 2026, to capture any newly published studies within the defined time frame. Newly identified studies were screened independently by two reviewers using the same eligibility criteria.

Search Strategy

The search strategy combined controlled vocabulary terms (e.g., Medical Subject Headings (MeSH) terms in PubMed) and free-text keywords related to POCUS and PIV access. Boolean operators (AND/OR) were used to refine the search, and the strategy was adapted for each database. The detailed search strings used in each database are provided in Table 2. Filters were applied to restrict results to human studies published between 2021 and 2025.

**Table 2:** Database Search Strategy

Database	Search Strategy
PubMed/MEDLINE	(“point-of-care ultrasound” OR “ultrasound-guided” OR POCUS) AND (“peripheral intravenous access” OR “peripheral venous cannulation” OR PIV) AND (“emergency department” OR trauma)
Scopus	TITLE-ABS-KEY (“point-of-care ultrasound” OR “ultrasound guided”) AND TITLE-ABS-KEY (“peripheral intravenous” OR “venous cannulation”) AND TITLE-ABS-KEY (“emergency department” OR trauma)
Web of Science	TS=(“point-of-care ultrasound” OR “ultrasound-guided”) AND TS=(“peripheral intravenous access” OR “venous cannulation”) AND TS=(“emergency department” OR trauma)
Embase	(‘point of care ultrasound’ OR ‘ultrasound guided’) AND (‘peripheral intravenous access’ OR ‘peripheral venous cannulation’) AND (‘emergency department’ OR trauma)

Selection Process

All identified records were exported into EndNote X21 for reference management (Clarivate, London, UK). Duplicate records were identified and removed using the software’s automated duplicate detection function, followed by manual verification to ensure accuracy. After duplicate removal, titles and abstracts were screened independently by two reviewers for eligibility. Potentially relevant studies underwent full-text review. Disagreements at any stage were resolved through discussion and consensus to minimize selection bias.

Data Collection Process

A standardized data extraction form was developed prior to data collection. Two reviewers independently extracted data from each included study. Extracted variables included study characteristics (author, year, country, study design, sample size), population characteristics, intervention and comparator details, operator experience, and outcome measures. Any discrepancies in data extraction were resolved through discussion and cross-verification with the original article.

Data Items

The primary data items extracted were the first-attempt success rate and the overall success rate of PIV cannulation. Secondary data items included time to successful cannulation, number of attempts, complication rates, requirement for alternative vascular access, and patient-centered outcomes. Additional contextual variables, such as operator training level and use of static versus dynamic ultrasound guidance, were also recorded to explore potential sources of heterogeneity.

Risk of Bias Assessment

The methodological quality of included RCTs was assessed using the Cochrane Risk of Bias 2 (ROB2) tool [11]. For non-randomized and observational studies, the Risk Of Bias In Non-randomized Studies of Interventions (ROBINS-I) tool was applied [12]. Two reviewers independently performed the risk of bias assessment. Discrepancies were resolved through consensus. The results of the quality assessment were incorporated into the interpretation of findings to ensure a balanced and critical synthesis of the evidence.

Synthesis of Results

A qualitative synthesis of findings was conducted. A meta-analysis was not performed due to substantial clinical and methodological heterogeneity among the included studies. Specifically, variations in patient populations (general emergency vs. trauma-specific vs. DIVA-only cohorts), differences in operator expertise and training, variability in ultrasound techniques (static vs. dynamic guidance), inconsistent outcome definitions (e.g., differing definitions of first-attempt success), and heterogeneity in reporting time-to-cannulation metrics limited the comparability of pooled estimates. Conducting a meta-analysis under these conditions could have produced misleading summary effect estimates and reduced the interpretability of results. Therefore, a structured narrative synthesis was undertaken to provide a clinically meaningful interpretation of the available evidence.

Reporting Bias Assessment

Where sufficient information was available, selective outcome reporting was considered during the risk of bias assessment. However, due to the limited number of homogeneous studies within comparable outcome categories, formal statistical assessment of publication bias was not performed.

Certainty Assessment

The overall strength and consistency of evidence were interpreted in light of study design, risk of bias, and consistency of findings across studies. Emphasis was placed on methodological rigor and reproducibility to inform clinical applicability in emergency and trauma settings.

Results

Study Selection Process

**Figure 1:**
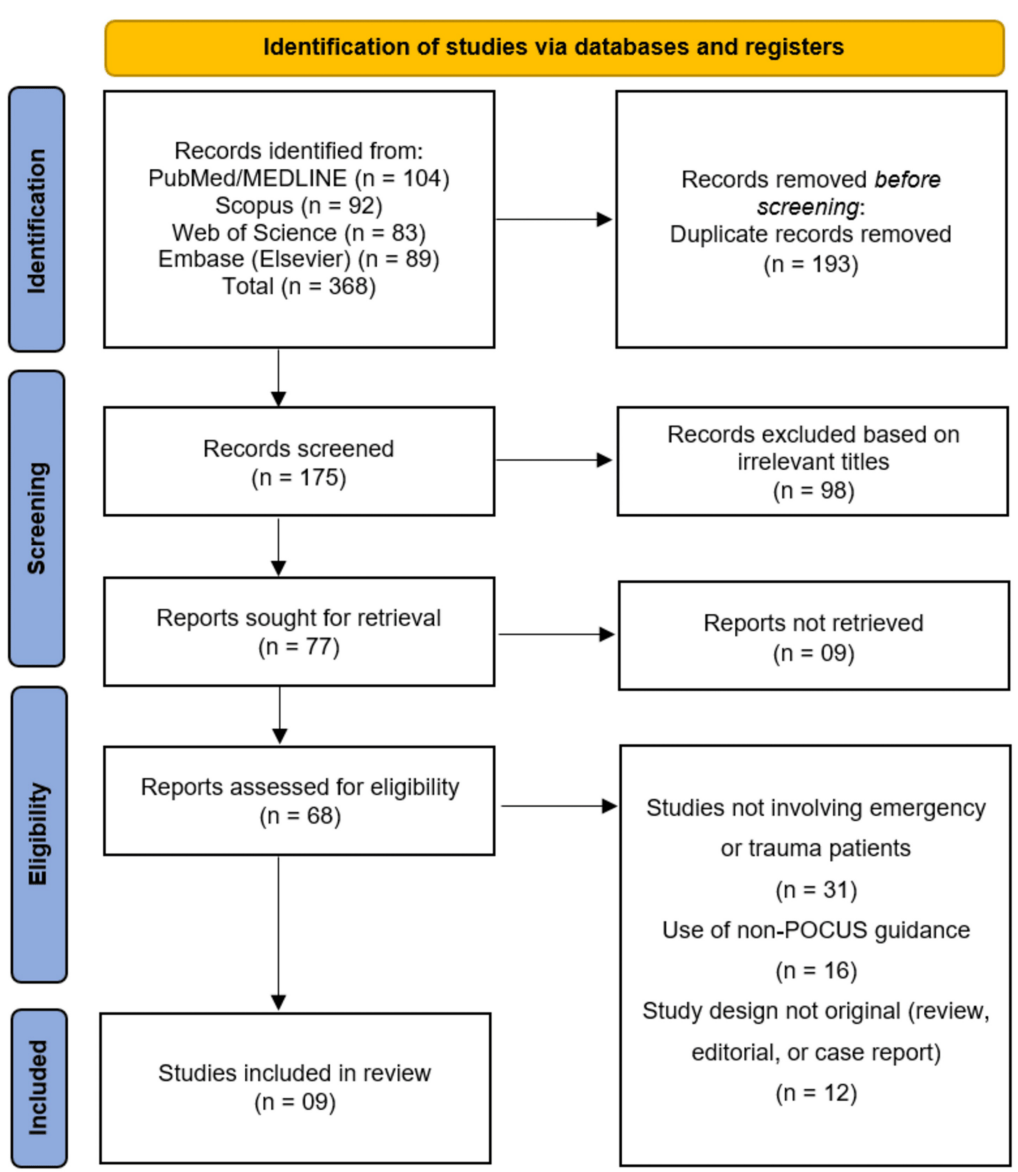
Studies Selection Process Illustrated in PRISMA Flowchart PRISMA: Preferred Reporting Items for Systematic Reviews and Meta-Analyses; POCUS: point-of-care ultrasound

The study selection process followed the PRISMA guidelines (Figure 1). A comprehensive literature search of four electronic databases - PubMed/MEDLINE, Scopus, Web of Science, and Embase (Elsevier) - yielded a total of 368 records. After the removal of 193 duplicate records, 175 unique studies remained for initial screening. Screening of titles and abstracts led to the exclusion of 98 records that were clearly irrelevant to the research question. The remaining 77 reports were sought for retrieval, of which nine could not be obtained. Attempts were made to access these full texts through institutional subscriptions and author contact; however, they remained unavailable, likely due to access limitations, and could not be included despite being potentially eligible based on abstract screening. A total of 68 full-text reports were assessed for eligibility against the predefined inclusion criteria. Of these, 59 reports were excluded for the following reasons: studies not involving emergency or trauma patients (n = 31), use of non-POCUS guidance methods (n = 16), and study designs that were not original research, including reviews, editorials, or case reports (n = 12). Following this rigorous selection process, nine studies met all inclusion criteria and were included in this systematic review [13-21].

Characteristics of Included Studies

A total of nine studies met the inclusion criteria for this systematic review evaluating the accuracy and effectiveness of POCUS for PIV access in emergency and trauma patients. The characteristics of these studies are summarized in Table 3. The included studies comprised four RCTs [13,21], one quasi-experimental study [17], two retrospective cohort studies [14,15], one prospective observational study [18], and one prospective pilot study [16]. The studies were conducted across diverse geographical settings, including Taiwan [13], China [14], the United States [15,17-19], Australia [16], Canada [20], and Italy [21]. Sample sizes ranged from 32 participants in a pilot study of intensive care paramedics [16] to 442 participants in a randomized trial of mono-plane versus bi-plane ultrasound approaches [21]. The clinical settings included emergency departments [14,15,19,21], medical centres [13], inpatient wards [17], trauma centres [18], pediatric emergency departments [20], and out-of-hospital settings with intensive care paramedic teams [16].

**Table 3:** Characteristics of Included Studies Evaluating POCUS-Guided PIV Access RCT: randomized controlled trial; ED: emergency department; IV: intravenous; PIV: peripheral intravenous; US: ultrasound; POCUS: point-of-care ultrasound; USGPIV: ultrasound-guided peripheral intravenous; DIVA: difficult intravenous access; RN/RNs: registered nurse(s); ICP/ICPs: intensive care paramedic(s); SBML: simulation-based mastery learning; MC: midline catheter; PICC: peripherally inserted central catheter; ITS: interrupted time series; QI: quality improvement; DT: dwell time; Obs.: observational; Exp.: experimental; Pts: patients; Yrs: years

Author (Year)	Country	Study Design	Setting	Sample Size	Population	Intervention	Comparator	Operator	Primary Outcomes
Kuo et al., [13] (2025)	Taiwan	RCT (crossover)	Medical center	36	Adults with difficult IV access (score 3-4)	US-guided PIV (2-person technique)	Traditional blind PIV	Trained ED nurses	First-attempt success, Overall success, Attempts
Yuan et al., [14] (2025)	China	Retrospective cohort (single-center)	ED	200 (102 vs 98)	Adults ≥18 yrs, DIVA	US-guided PIV	Landmark method	Trained RNs (≥2 yrs exp.)	First-attempt success
Malik et al., [15] (2023)	USA	Retrospective	Tertiary ED	388	Adults >18, failed IV	US-guided PIV	None	Trained RNs	Catheter survival, DT, failure
Burton et al., [16] (2023)	Australia	Prospective pilot	3 ICP teams (rural & metro)	32	Adults ≥18, DIVA	POCUS IV	None	ICPs	Success rate, first-attempt success
Amick et al., [17] (2022)	USA	Quasi-exp	Inpatient, tertiary	148	DIVA patients	USGPIV SBML	Pre-training/pilot	Nurses	↓ MC & PICC use, ITS effect
McKinley et al., [18] (2024)	USA	Prospective obs.	Level I trauma ED	16 nurses/200 attempts	ED pts (DIVA & non-DIVA)	POCUS IV	None	Trained ED nurses	USGPIV success, attempts to credentialing, training time, failure causes
Kalam et al., [19] (2023)	USA	Prospective QI	ED, single hospital	104	Adult sepsis	Nurse POCUS	Physician assessment	Trained nurses	Physician agreement, fluid changes, confidence, accuracy
Jamal et al., [20] (2023)	Canada	Pilot	Tertiary pediatric ED	210	Pediatric, difficult IV	US-guided PIV	Standard PIV	12 trained nurses	First-attempt success, overall success, attempts, time, calls
Baion et al., [21] (2023)	Italy	RCT, single-center, non-blinded	ED, Molinette Hospital, Turin	442	Adults with difficult IV access	POCUS PIV (mono- or bi-plane)	Mono-plane vs Bi-plane	38 operators (novice/intermediate/expert; RNs, residents, physicians)	First attempt success

The populations studied predominantly consisted of adults with DIVA, with three studies specifically enrolling patients with difficult venous access [13,14,16] and one study focusing on patients with prior failed attempts [21]. One study examined pediatric patients with difficult access [20], while another evaluated patients with sepsis in the emergency department [19]. The operators performing ultrasound-guided PIV access included trained emergency nurses [13-15,18-20], intensive care paramedics [16], and a mix of nurses, residents, and physicians [21]. Primary outcomes varied across studies but consistently included first-attempt success rates [13,14,16,20,21], overall success rates [13,15,18,20], and procedural time metrics [13,14,16].

First-Attempt Success Rates

First-attempt success rates for ultrasound-guided PIV access were consistently superior to conventional landmark techniques across the included studies, as detailed in Table 4. In the randomized crossover trial by Kuo and colleagues, ultrasound-guided insertion achieved a first-attempt success rate of 63.89% compared to only 13.89% with traditional blind insertion, yielding an odds ratio of 10.97 (95% confidence interval 3.43 to 35.13) [13]. Similarly, Yuan and colleagues reported a first-attempt success rate of 84.3% with ultrasound guidance versus 51.0% with the landmark method, corresponding to a relative risk of approximately 1.65 (95% confidence interval 1.31 to 2.08) [14].

**Table 4:** Comparative Effectiveness and Accuracy Outcomes of POCUS-Guided Versus Conventional PIV Access OR: odds ratio; RR: relative risk; CI: confidence interval; MD: mean difference; NR: not reported; POCUS: point-of-care ultrasound; DIVA: difficult intravenous access; USGPIV: ultrasound-guided peripheral intravenous; PIV: peripheral intravenous; SBML: simulation-based mastery learning; MC: midline catheter; Mono: monoplane ultrasound guidance; Bi: biplane ultrasound guidance; mm: millimeter; min: minutes

Author (Year)	First-Attempt Success (%) POCUS	First-Attempt Success (%) Control	Overall Success (%)	Mean Attempts (POCUS vs Control)	Time to Cannulation (Minutes)	Complications (%)	Effect Size (RR/OR, 95% CI)
Kuo et al., [13] (2025)	63.89%	13.89%	88.89% vs 13.89%	1.36 vs 1.86	3.62 vs 2.37 (total time); 2.68 vs 1.38 (first attempt)	11.11% vs 8.33%	First-attempt: OR 10.97 (95% CI 3.43-35.13); Overall success: OR 49.60 (95% CI 12.18-202.05); Complications: RR 1.33 (95% CI 0.32-5.54); Mean attempts: MD -0.50 (95% CI -0.70 to -0.30)
Yuan et al., [14] (2025)	84.3% (86/102)	51.0% (50/98)	NR	1.3 ± 0.5 vs 2.4 ± 1.1	6.7 ± 2.3 vs 11.2 ± 3.9	5.9% vs 16.3%	RR ≈ 1.65 (1.31-2.08)
Malik et al., [15] (2023)	NR	NR	980/1190 ≈ 82.4%	NR	NR	NR	NR
Burton et al., [16] (2023)	First-Attempt Success	87%	NR	50%	1.5	NR	NR
Amick et al., [17] (2022)	USGPIV SBML Training Study	NR	NR	NR	NR	NR	MC utilization decreased from 1.86 to 1.33 per 1000 patient-days
McKinley et al., [18] (2024)	80% (training), 92.9% (post-credentialing); DIVA 67.9–100%	NR	92.9%	Median 11 attempts to achieve 10 successes	NR	NR	NR
Kalam et al., [19] (2023)	NR	NR	NR	NR	NR	NR	NR
Jamal et al., [20] (2023)	86.5%	NR	91.9%	1.1 vs NR	NR	NR	NR
Baion et al., [21] (2023)	70.8% (Mono 68.3%, Bi 73.3%)	- (all had prior failed attempts)	100%	~1.30	0.67	0	Bi-plane vs mono-plane OR 0.97 (0.48-1.97); vessel diameter ≥3.5 mm OR 2.88 (1.39-5.96)

In the pediatric emergency department setting, Jamal and colleagues demonstrated a first-attempt success rate of 86.5% following implementation of a comprehensive ultrasound-guided PIV training programme [20]. Burton and colleagues, in their out-of-hospital pilot study with intensive care paramedics, reported first-attempt success rates of 87% [16]. The randomized trial by Baion and colleagues, which compared mono-plane and bi-plane ultrasound approaches in patients with prior failed attempts, found first-attempt success rates of 68.3% for the mono-plane technique and 73.3% for the bi-plane technique, with an odds ratio of 0.97 (95% confidence interval 0.48 to 1.97) for the comparison between techniques [21]. Notably, vessel diameter of 3.5 millimetres or greater was associated with significantly higher success (odds ratio 2.88, 95% confidence interval 1.39 to 5.96) [21]. McKinley and colleagues observed that emergency nurses achieved first-attempt success rates of 80% during training and 92.9% following credentialing, with success rates ranging from 67.9% to 100% in patients with difficult access [18].

Overall Success Rates and Procedural Attempts

Overall success rates for ultrasound-guided PIV access were also markedly improved compared to conventional techniques. Kuo and colleagues reported overall success rates of 88.89% with ultrasound guidance compared to only 13.89% with traditional insertion, representing an odds ratio of 49.60 (95% confidence interval 12.18 to 202.05) [13]. The number of insertion attempts was significantly reduced with ultrasound guidance, with a mean difference of -0.50 attempts (95% confidence interval -0.70 to -0.30) in favour of ultrasound [13]. Yuan and colleagues similarly found that ultrasound guidance required fewer attempts (1.3 ± 0.5) compared to the landmark method (2.4 ± 1.1) [14]. In a large retrospective cohort, Malik and colleagues documented an overall success rate of 82.4% (980 out of 1190 catheter placements) with ultrasound guidance in the emergency department setting [15]. McKinley and colleagues reported that emergency nurses required a median of 11 attempts to achieve 10 successful ultrasound-guided insertions during their credentialing process [18]. In the pediatric population, Jamal and colleagues achieved an overall success rate of 91.9%, with a mean of 1.1 attempts per successful insertion [20]. Baion and colleagues reported 100% overall success in their trial, though this was in a population where all patients had prior failed attempts, and the mean number of attempts was approximately 1.30 [21].

Time to Cannulation

Procedural time metrics varied across studies, with some reporting shorter times for ultrasound guidance and others noting longer preparation or total procedure times. Kuo and colleagues reported a mean total procedure time of 3.62 minutes for ultrasound-guided insertion compared to 2.37 minutes for the traditional method, while first-attempt cannulation time was 2.68 minutes versus 1.38 minutes, respectively [13], noting that ultrasound-guided insertion inherently includes additional steps such as probe preparation, gel application, machine setup, and vein scanning. Yuan and colleagues found that ultrasound guidance required a mean time of 6.7 ± 2.3 minutes compared to 11.2 ± 3.9 minutes for the landmark method, suggesting a time benefit for ultrasound when considering overall procedural duration, including multiple attempts [14]. Burton and colleagues reported a median time to cannulation of 1.5 minutes in their paramedic-led pilot study [16]. Baion and colleagues documented a mean time to cannulation of 0.67 minutes in their randomized trial, reflecting the experience of operators with mixed skill levels [21].

Complications and Safety Outcomes

Complication rates were generally low across studies and comparable between ultrasound-guided and conventional techniques. Kuo and colleagues reported complication rates of 11.11% with ultrasound guidance versus 8.33% with traditional insertion, yielding a relative risk of 1.33 (95% confidence interval 0.32 to 5.54), indicating no statistically significant difference in safety outcomes [13]. Yuan and colleagues observed lower complication rates with ultrasound guidance (5.9%) compared to the landmark method (16.3%) [14]. Baion and colleagues reported no complications in their trial of mono-plane and bi-plane ultrasound techniques [21]. The remaining studies either did not report complication rates as primary outcomes or did not identify significant safety concerns associated with ultrasound-guided PIV access [15-20].

Operator Training and Credentialing Outcomes

Several studies evaluated the impact of training and credentialing on the effectiveness of ultrasound-guided PIV access. McKinley and colleagues prospectively observed emergency nurses undergoing a credentialing programme and found that first-attempt success improved from 80% during training to 92.9% post-credentialing, with a median of 11 attempts required to achieve competency [18]. The most common causes of failure included vein collapse, hematoma formation, and inability to visualize the needle tip [18]. Amick and colleagues implemented a structured ultrasound-guided PIV catheter insertion training programme and demonstrated a significant reduction in midline catheter utilization, decreasing from 1.86 to 1.33 per 1000 patient-days, suggesting improved peripheral access success and reduced need for more invasive alternatives [17]. Kalam and colleagues evaluated nurse-performed POCUS in septic emergency department patients and reported high levels of physician agreement with ultrasound findings, increased clinician confidence, and appropriate fluid management changes based on ultrasound assessment [19]. Jamal and colleagues described a comprehensive training programme in a pediatric emergency department, which resulted in successful implementation of ultrasound-guided PIV insertion with high first-attempt and overall success rates among trained nurses [20]. Burton and colleagues demonstrated that intensive care paramedics could be trained to perform ultrasound-guided PIV access in out-of-hospital settings with acceptable success rates [16]. The randomized trial by Baion and colleagues included operators of varying experience levels - novice, intermediate, and expert - and found that operator experience did not significantly affect first-attempt success when comparing mono-plane and bi-plane techniques [21].

Risk of Bias Assessment Results

The risk of bias for the two RCTs was assessed using the RoB-2 tool (Table 5). Kuo and colleagues demonstrated low risk of bias across all domains, resulting in an overall low risk judgement [13]. Baion and colleagues were assessed as low risk in four domains, with some concerns identified in the measurement of the outcome domain due to the lack of blinding inherent to device-based interventions, leading to an overall judgement of some concerns [21].

**Table 5:** Risk of Bias Assessment for Randomized Controlled Trials (RoB 2) RoB 2: Risk of Bias 2

Author (Year)	Randomization Process	Deviations from Intended Interventions	Missing Outcome Data	Measurement of the Outcome	Selection of the Reported Result	Overall Risk of Bias
Kuo et al., [13] (2025)	Low	Low	Low	Low	Low	Low
Baion et al., [21] (2023)	Low	Low	Low	Some concerns	Low	Some concerns

The risk of bias for the seven non-randomized studies was assessed using the ROBINS-I tool (Table 6). Five studies were judged to have low risk of bias overall, including the retrospective cohort studies by Yuan and colleagues [14] and Malik and colleagues [15], the prospective pilot study by Burton and colleagues [16], the prospective observational study by McKinley and colleagues [18], and the prospective pilot study by Jamal and colleagues [20]. Two studies were assessed as having a moderate risk of bias overall. The quasi-experimental study by Amick and colleagues received moderate risk ratings in the domains of deviations from intended interventions and measurement of outcomes [17]. The prospective quality improvement study by Kalam and colleagues was rated as moderate risk in the domains of deviations from intended interventions and measurement of outcomes due to lack of standardization and subjective outcome measures [19]. No studies were judged to be at critical or serious risk of bias.

**Table 6:** Risk of Bias Assessment for Non-randomized Studies (ROBINS-I) ROBINS-I: Risk Of Bias In Non-randomized Studies - of Interventions

Author (Year)	Study Design	Domain 1: Confounding	Domain 2: Selection of Participants	Domain 3: Classification of Interventions	Domain 4: Deviations from Intended Interventions	Domain 5: Missing Data	Domain 6: Measurement of Outcomes	Domain 7: Selection of Reported Result	Overall Risk of Bias
Yuan et al., [14] (2025)	Retrospective cohort	Low	Low	Low	Low	Low	Low	Low	Low
Malik et al., [15] (2023)	Retrospective cohort	Low	Low	Low	Low	Low	Low	Low	Low
Burton et al., [16] (2023)	Prospective pilot	Low	Low	Low	Low	Low	Low	Low	Low
Amick et al., [17] (2022)	Quasi-experimental	Low	Low	Low	Moderate	Low	Moderate	Low	Moderate
McKinley et al., [18] (2024)	Prospective observational	Low	Low	Low	Low	Low	Low	Low	Low
Kalam et al., [19] (2023)	Prospective QI	Low	Low	Low	Moderate	Low	Moderate	Low	Moderate
Jamal et al., [20] (2023)	Prospective pilot	Low	Low	Low	Low	Low	Low	Low	Low

Discussion

This systematic review synthesizes evidence from nine studies evaluating the accuracy and effectiveness of POCUS for PIV access in emergency and trauma patients. The findings demonstrate that ultrasound-guided PIV access significantly improves first attempt and overall success rates compared to conventional landmark techniques, particularly in patients with DIVA. These benefits are accompanied by reduced numbers of insertion attempts and, in some studies, shorter overall procedural times when accounting for multiple attempts. The evidence also supports the feasibility of training diverse operator groups - including emergency nurses, intensive care paramedics, and physicians - to achieve proficiency in ultrasound-guided PIV access. Patient outcomes in the included studies were defined as procedural success, complication rates, need for alternative vascular access, catheter dwell time, pain scores, and patient satisfaction; mortality was not assessed as an outcome, as it is not directly influenced by the vascular access method. These improvements consequently reduce the need for more invasive alternatives such as midline catheters.

The most striking finding across the included studies is the substantial improvement in first-attempt success rates with ultrasound guidance. Kuo and colleagues reported an odds ratio of 10.97 for first-attempt success with ultrasound compared to traditional blind insertion, while Yuan and colleagues demonstrated a relative risk of approximately 1.65 [13,14]. These effect sizes are clinically meaningful, as multiple insertion attempts are associated with patient discomfort, delayed treatment, and increased risk of complications. The magnitude of benefit observed in this review is consistent with previous systematic reviews examining ultrasound-guided vascular access. A meta-analysis by Egan and colleagues reported that ultrasound guidance for PIV access significantly increased first-attempt success rates (risk ratio 1.71, 95% confidence interval 1.45 to 2.02) and overall success rates (risk ratio 1.30, 95% confidence interval 1.13 to 1.49) compared to traditional methods [22]. Similarly, a Cochrane review by Brass and colleagues examining ultrasound-guided versus landmark method for PIV cannulation found moderate-certainty evidence that ultrasound reduces the number of attempts and improves success rates in adults with difficult access [23]. The consistency of findings across multiple systematic reviews strengthens the evidence base supporting routine use of ultrasound in patients with predicted DIVA.

The success rates observed in pediatric populations warrant specific consideration. Jamal and colleagues achieved a first-attempt success rate of 86.5% and an overall success rate of 91.9% following implementation of a comprehensive training programme in a pediatric emergency department [20]. These findings are particularly important given that pediatric patients present unique challenges for PIV access, including smaller vessel calibre and reduced cooperation. The success rates achieved by Jamal and colleagues exceed these pooled estimates, possibly reflecting the benefits of a structured training programme and dedicated pediatric practice [20]. This suggests that ultrasound guidance may be particularly valuable in pediatric emergency settings where difficult access is common and repeated attempts are poorly tolerated.

The finding that vessel diameter of 3.5 millimetres or greater was associated with significantly higher success (odds ratio 2.88) in the trial by Baion and colleagues highlights an important practical consideration for clinicians [21]. This observation aligns with previous research by Witting and colleagues, who demonstrated that vein diameter measured by ultrasound is a strong predictor of successful cannulation, with veins smaller than 0.4 centimetres being associated with significantly lower success rates [24]. These findings underscore the importance of preoperative ultrasound assessment to identify optimal vessels and suggest that clinicians should consider alternative approaches when only small-calibre veins are available, even with ultrasound guidance.

The time to cannulation outcomes presents an interesting dichotomy. While Kuo and colleagues reported longer total procedure times for ultrasound-guided insertion (3.62 vs. 2.37 minutes) [13], Yuan and colleagues found that ultrasound guidance required less total time when accounting for multiple attempts (6.7 vs. 11.2 minutes) [14]. This apparent discrepancy likely reflects the difference between first-attempt cannulation time and total procedural time, including multiple attempts. When initial attempts fail with traditional methods, the cumulative time required for multiple attempts and the need to involve additional operators can substantially prolong the overall procedure. A prospective study by Costantino and colleagues similarly reported that while ultrasound-guided insertion took longer for the initial attempt, the total procedure time was significantly shorter when accounting for the high failure rate of traditional methods [25]. From a clinical perspective, the most relevant metric is probably the time to successful cannulation, which favours ultrasound guidance in patients with difficult access where multiple attempts would otherwise be required.

The complication rates reported across included studies were generally low and comparable between ultrasound-guided and conventional techniques. Kuo and colleagues found no significant difference in complication rates (relative risk 1.33, 95% confidence interval 0.32 to 5.54) [13], while Yuan and colleagues observed lower complication rates with ultrasound guidance (5.9% vs. 16.3%) [14]. Baion and colleagues reported no complications in their trial [21]. The safety profile of ultrasound-guided PIV access appears favourable, likely because improved visualization reduces the risk of posterior wall puncture, arterial puncture, and multiple traumatic attempts. However, it should be noted that most studies were not adequately powered to detect differences in rare but serious complications such as nerve injury or infection.

The operator training and credentialing outcomes reported in several studies provide valuable insights for clinical implementation. McKinley and colleagues demonstrated that emergency nurses required a median of 11 attempts to achieve competency, with first-attempt success improving from 80% during training to 92.9% following credentialing [18]. This learning curve is similar to that reported by Stolz and colleagues, who found that emergency physicians required approximately 10 to 15 supervised ultrasound-guided PIV insertions to achieve consistent proficiency [26]. Amick and colleagues demonstrated that implementation of an ultrasound-guided PIV training programme was associated with a significant reduction in midline catheter utilization, decreasing from 1.86 to 1.33 per 1000 patient-days [17]. This finding has important implications for healthcare resource utilization and patient experience, as midline catheters and peripherally inserted central catheters are more invasive, more expensive, and associated with additional complications compared to PIV catheters.

The finding by Baion and colleagues that operator experience did not significantly affect first-attempt success when comparing mono-plane and bi-plane techniques is noteworthy [21]. This suggests that either technique can be effectively taught to operators of varying experience levels, and that the choice between mono-plane and bi-plane approaches may be less important than ensuring adequate training and supervision. However, a systematic review by Gottlieb and colleagues examining ultrasound-guided vascular access techniques found that dynamic approaches (where the needle is visualized in real-time) were associated with higher success rates than static techniques, regardless of operator experience [27]. The present review did not directly compare dynamic and static techniques, but all included studies employed real-time ultrasound guidance, which is consistent with current best practice recommendations.

The diversity of operator types successfully trained to perform ultrasound-guided PIV access across included studies, including emergency nurses [13-15,18-20], intensive care paramedics [16], and physicians [21], supports a multidisciplinary approach to implementation. This is particularly relevant in emergency and trauma settings where multiple clinician groups may be involved in patient care. The findings of Kalam and colleagues regarding nurse-performed POCUS in septic patients extend these benefits beyond vascular access to include diagnostic assessment and fluid management decisions [19]. This suggests that training emergency nurses in POCUS may have broader benefits for patient care beyond improved intravenous access.

Several of the included studies provide evidence for the effectiveness of ultrasound-guided PIV access in specific patient populations. Burton and colleagues demonstrated feasibility in out-of-hospital settings with intensive care paramedics [16], which has implications for prehospital care where difficult access is common and alternative options are limited. A study by Shi and colleagues similarly reported that paramedics could be trained to perform ultrasound-guided PIV access in the prehospital setting with success rates exceeding 80% [28]. Malik and colleagues provided real-world effectiveness data from a large retrospective cohort, with an overall success rate of 82.4% in emergency department patients with prior failed attempts [15]. This pragmatic evidence complements the findings from randomized trials and supports the generalizability of ultrasound-guided PIV access to routine clinical practice.

Despite the consistent evidence supporting ultrasound-guided PIV access, several barriers to implementation should be acknowledged. These include the need for equipment availability, training costs, and time required for competency attainment. However, the study by Amick and colleagues suggests that investment in training may be offset by reduced utilization of more expensive vascular access devices [17]. Future research should include formal health economic evaluations to confirm these findings across different healthcare settings.

The included studies also highlight the importance of ongoing quality assurance and monitoring of outcomes following implementation of ultrasound-guided PIV access programmes. McKinley and colleagues identified common causes of failure, including vein collapse, hematoma formation, and inability to visualize the needle tip [18]. These findings can inform training curricula and competency assessment. A study by Weiner and colleagues described a structured credentialing process for ultrasound-guided PIV access that included didactic education, simulation-based training, and supervised clinical insertions, with demonstrated improvements in success rates and reductions in complications [29]. Such structured approaches are likely to be more effective than informal training and should be considered by institutions implementing ultrasound-guided PIV access programmes.

Limitations

This systematic review has several limitations that should be considered when interpreting the findings. First, the included studies exhibited methodological heterogeneity in terms of study design, patient populations, operator experience, and outcome definitions. Although statistical heterogeneity (I^2^) was not formally quantified due to the small number of studies and outcome variability, a meta-analysis was not performed; narrative synthesis was used instead. While all studies addressed ultrasound-guided PIV access, variations in technique (mono-plane vs. bi-plane) and setting (emergency department, out-of-hospital, mixed adult-pediatric cohorts) limit direct comparability of effect estimates. All included studies were comparative, consistent with eligibility criteria; no study lacked a comparator, addressing conceptual consistency and internal validity concerns. Second, the risk of bias assessment revealed that two non-randomized studies were at moderate risk of bias [17,19], and one randomized trial had some concerns due to a lack of blinding [21]. Operator experience and patient severity were key potential confounders, and baseline characteristics were generally balanced in the RCTs. While blinding of operators is inherently challenging in device-based interventions, outcome assessors were sometimes not blinded, and first-attempt success definitions varied across studies, which may have introduced performance or detection bias. Third, publication bias cannot be excluded. Although the consistent direction of effect was observed, this may reflect small-study effects, selective publication, or shared methodological biases; a formal funnel plot or regression-based assessment was not feasible due to the limited number of studies. Fourth, the generalizability of findings to all emergency and trauma settings may be limited, as most studies were conducted in tertiary or academic centers with access to ultrasound equipment and trained operators. The effectiveness of ultrasound-guided PIV access in resource-limited settings or smaller community hospitals remains less certain. Fifth, the included studies predominantly focused on short-term outcomes such as first-attempt success and time to cannulation, with limited data on longer-term outcomes, including catheter survival, complications beyond the immediate procedure, and patient-reported outcomes such as pain and satisfaction. Sixth, the definition of DIVA varied across studies, with some using validated scoring systems [13] and others using clinical judgment or prior failed attempts [14,15,21]. This variability may have influenced the observed effect sizes. Finally, the small number of studies in specific subgroups (mixed pediatric-adult cohorts, out-of-hospital settings, septic patients) limits the strength of conclusions for these populations, despite a generally consistent direction of effect.

## Conclusions

This systematic review provides evidence that POCUS-guided PIV access is consistently associated with higher first-attempt and overall success rates compared to conventional landmark techniques in emergency and trauma patients with difficult access. The benefits extend to fewer insertion attempts, while findings on procedural times were mixed, with some studies showing longer first-attempt times but shorter total times when accounting for multiple attempts. Complication rates were generally comparable to or lower than traditional methods; however, definitions of complications varied, studies were not always powered to detect differences, and follow-up was predominantly immediate rather than longitudinal. Training programmes for diverse operator groups, including emergency nurses, intensive care paramedics, and physicians, demonstrated high success in some studies, but effectiveness depended on structured curricula, supervised insertion volume, and institutional support, and reductions in invasive device use were reported inconsistently across studies. A vessel diameter of 3.5 millimetres or greater was generally associated with higher success, highlighting the potential value of pre-procedure ultrasound assessment. While the evidence base includes both randomized and non-randomized studies with predominantly low to moderate risk of bias, the limited number of studies, mixed designs, lack of formal Grading of Recommendations Assessment, Development and Evaluation (GRADE) assessment, and absence of health economic analyses preclude recommending routine use with high certainty, although findings support consideration of ultrasound guidance as a beneficial adjunct in patients with predicted or proven difficult access in emergency and trauma care. Future research should focus on standardizing outcome definitions, conducting health economic evaluations, and examining longer-term outcomes, including catheter survival and patient-reported experience measures, and implementation efforts should include structured training and credentialing programmes, quality assurance monitoring, and strategies to address barriers to adoption, particularly in resource-limited settings.
